# Diagnostic Clinical Predictors of Early Recovery from Stone-Induced Systemic Inflammatory Response Syndrome After Urgent Decompression

**DOI:** 10.3390/diagnostics15172282

**Published:** 2025-09-08

**Authors:** Sungbin Yoon, Yeonuk Jung, Han Kyu Chae, Wook Nam, Hoon Yu, Youngjong Cho, Sung Jin Kim

**Affiliations:** 1 Department of Nephrology, Gangneung Asan Hospital, University of Ulsan College of Medicine, Gangneung 25440, Republic of Korea; d230627@gnah.co.kr (S.Y.); hoon2345@ulsan.ac.kr (H.Y.); 2 Department of Urology, Asan Medical Centre, University of Ulsan College of Medicine, Seoul 05505, Republic of Korea; wowoei11@gmail.com; 3 Department of Urology, Gangneung Asan Hospital, University of Ulsan College of Medicine, Gangneung 25440, Republic of Korea; hanqsinopoli@ulsan.ac.kr (H.K.C.); wooki6258@gnah.co.kr (W.N.); 4 Department of Radiology, Gangneung Asan Hospital, University of Ulsan College of Medicine, Gangneung 25440, Republic of Korea; ohggamja@ulsan.ac.kr

**Keywords:** systemic inflammatory response syndrome, obstructive pyelonephritis, retrograde ureteral stenting, percutaneous nephrostomy, sex disparity, systemic susceptibility

## Abstract

**Background**: To identify clinical predictors of early recovery in patients with stone-induced systemic inflammatory response syndrome (SIRS) undergoing emergency decompression and compare the short-term inflammatory and renal function outcomes between retrograde ureteral stenting (RUS) and percutaneous nephrostomy (PCN). **Method**: We retrospectively evaluated data from 178 patients with stone-induced SIRS who were treated with RUS (*n* = 98) or PCN (*n* = 80) between 2011 and 2020. Early recovery was defined as readiness for discharge or no fever relapse within 3 days after drainage. **Results**: Univariate and multivariate logistic regression analyses identified significant predictors, and clinical outcomes were compared based on drainage methods. Univariate analysis showed that diabetes mellitus (*p* = 0.009), mid (*p* = 0.014) and upper (*p* = 0.017) stone locations, stone size of 10–20 mm, and renal stones were associated with early recovery, whereas female sex (*p* = 0.01) predicted poorer outcomes. In multivariate analysis, diabetes mellitus (*p* = 0.031), as well as mid (*p* = 0.007) and upper (*p* = 0.026) stone locations, remained favorable predictors, and female sex (*p* = 0.036) remained a negative predictor. PCN was associated with a transient increase in leukocyte count but facilitated earlier creatinine recovery compared with RUS. **Conclusions**: Female sex was an independent predictor of failure to achieve early recovery after urgent decompression, whereas diabetes mellitus and proximal ureteral stone location were independent predictors of early recovery. Baseline clinical factors were the main determinants of early recovery, supporting management tailored to these factors.

## 1. Introduction

An increased urolithiasis incidence of 8% in men and 3% in women is largely attributed to the growing incidence of metabolic syndrome and global climatic changes [1,2]. The previous decade witnessed a two-fold surge in urinary tract obstructions caused by stones, often accompanied by infections [3]. Such obstructions can progress to life-threatening sepsis, which accounts for 10% of severe sepsis cases [4,5]. Moreover, urosepsis-related complications have increased mortality rates related to ureteroscopy for ureteric calculi in the past decade [6,7]. Therefore, research on the pathogenesis of systemic infectious conditions arising from urinary tract infections (UTIs) is crucial.

Systemic inflammatory response syndrome (SIRS) caused by calculus obstruction is a complex event arising from the convergence of multiple clinical conditions [8]. Its pathogenesis includes sudden-onset obstructive pyelonephritis (OPN) and systemic symptoms triggered by urine flow blockage by stones, enabling bacterial infiltration into the renal parenchyma [5]. Factors facilitating recovery following drainage are closely related to the pathogenesis of OPN leading to SIRS and offer insights into the influence of the renal system and systemic vulnerability. Immediate drainage of the renal system, antibiotic therapy, and fluid replacement are well-established treatments [5,9,10]. However, evidence is lacking regarding the relative effectiveness of two drainage methods (retrograde ureteral stenting [RUS] and percutaneous nephrostomy [PCN]) for infection control and renal function recovery [11,12,13]. Therefore, finding a clinical correlation may serve as an important basis for the early prevention of OPN progression to SIRS and effective SIRS management, contributing to the prevention of further development into septic shock.

In this study, we aimed to investigate the recovery patterns according to drainage methods and predictive factors for recovery in patients with SIRS. The study identified significant predictive factors with clinical relevance for recovery and provided insights into the differential impacts of RUS and PCN on recovery patterns.

## 2. Materials and Methods

### 2.1. Study Protocol

This retrospective study analyzed the medical records of patients with UTI due to upper urinary tract obstruction from urinary stones, who underwent emergency drainage at Gangneung Asan Hospital between January 2011 and December 2020. This study was approved by the Institutional Review Board of Gangneung Asan Hospital (IRB No. GNAH 2022-02-006, 7 March 2022). This study followed the tenets of the Declaration of Helsinki. The requirement for informed consent was waived owing to the retrospective nature of the study.

### 2.2. Study Population

**Figure 1:**
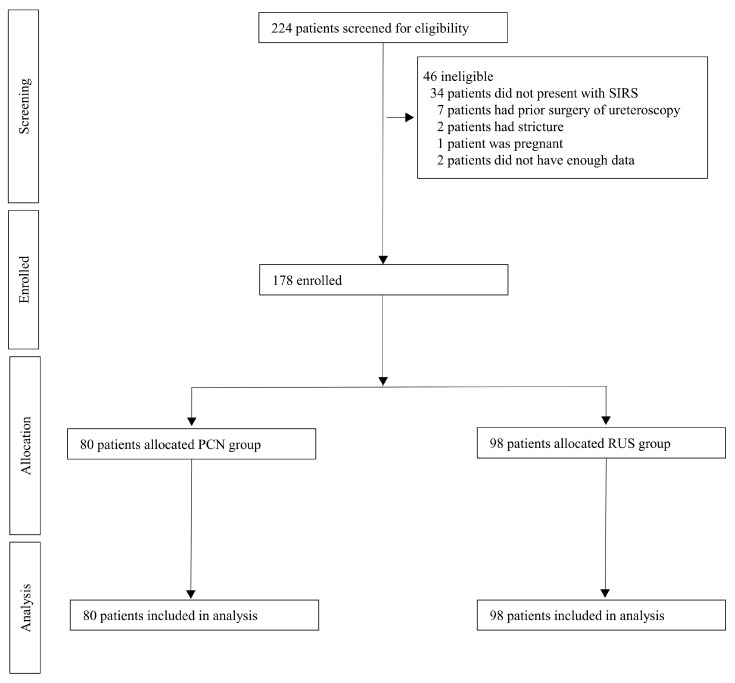
Patient selection flow chart.

Overall, 224 patients aged >20 years who underwent RUS or PCN procedures to treat computed tomography diagnosed urinary obstruction were screened for eligibility (Figure 1). Patients were excluded if they (1) did not present with SIRS at diagnosis (defined as ≥2 of the following: temperature >38 °C or <36 °C; heart rate >90 beats/min; respiratory rate >20 breaths/min or partial pressure of CO_2_ <32 mmHg; white blood cell [WBC] count >12,000, <4000 cells/μL, or with >10% immature forms), (2) had prior surgery (including reimplantation, ileal ureter replacement, urinary diversion, or kidney transplantation), (3) had urethral or ureteral strictures, (4) were pregnant, and (5) had insufficient clinical data. Of the 224 patients screened, 46 were deemed ineligible for the following reasons: 34 patients did not present with SIRS, 7 patients had undergone prior ureteroscopic surgery, 2 patients had urethral or ureteral strictures, 1 patient was pregnant, and 2 patients had insufficient clinical data. As a result, 178 patients were enrolled and subsequently allocated into two groups. 80 patients were assigned to the PCN group, and 98 patients to the RUS group.

SIRS, Systemic inflammatory response syndrome; PCN, Percutaneous nephrostomy; RUS, Retrograde ureteral stent

Clinical indicators (length of hospitalization; body temperature; age; sex; body mass index [BMI]; comorbidities (hypertension, diabetes mellitus [DM], stroke); interval from symptom presentation to drainage; and stone location, size, and laterality) and laboratory data (WBC count, absolute neutrophil count [ANC], C-reactive protein [CRP] levels, and serum creatinine [Cr] levels) were included in the analysis.

### 2.3. Antibiotic Protocol

Empirical antibiotics were initiated at presentation with SIRS concurrently with urgent decompression. First-line agents were third-generation cephalosporins or quinolones in clinically stable patients. Carbapenems were used upfront or escalated when initial vital signs were unstable (severe sepsis/septic shock) or ESBL risk (e.g., prior ESBL) was evident. De-escalation was performed according to culture/susceptibility results. Intravenous-to-oral switch and total duration were individualized based on fever resolution, hemodynamic stability, and improvement in inflammatory markers. Patients who underwent definitive surgery continued antibiotic therapy until the time of surgery.

### 2.4. Intervention

RUS was performed under local anesthesia and fluoroscopic guidance by a urology-trained clinician using a 6-Fr, 24 cm double-J stent. Pain control included analgesics such as pethidine, nonsteroidal anti-inflammatory drugs, and Tramadol. PCN was carried out by radiologists with fluoroscopic and ultrasound guidance, using an 8.5-Fr catheter in the prone position.

### 2.5. Outcomes

The primary outcome was ‘early recovery’ after drainage (based on the resolution of fever and patients’ readiness for discharge). Fever resolution was determined when patients’ peak temperature was <37.5 °C. Readiness for discharge was defined as the patient’s readiness to return home safely. Early recovery was characterized by the occurrence of either fever resolution or readiness for discharge within 3 days.

Secondary outcomes were established based on the recovery of laboratory values according to the drainage method. The assessment was based on significant improvements in inflammation-related indicators (WBC count, ANC, CRP levels, and kidney function-related indicators [Cr]) after drainage.

### 2.6. Statistical Analysis

Categorical variables were presented as percentages and analyzed using Pearson’s χ^2^ or Fisher’s exact test, while continuous variables were presented as mean ± standard deviation or medians with interquartile ranges after assessing normality distribution. Continuous variables were assessed using the Mann–Whitney U or Kruskal–Wallis’s test.

To evaluate the clinical significance of various factors and independently ascertain their predictive value, a univariate binary logistic regression analysis was performed, focusing on early recovery as the outcome. Variables with missing data were omitted from the analysis of study. For the multivariate model, only variables that could be assessed by clinicians and were suitable for external validation were included. In the multivariate analysis, all variables showing a significance of *p* < 0.1 in the univariate analysis were incorporated. The Akaike Information Criterion served to evaluate the model in a stepwise approach, sequentially removing each variable from the model under review. The model with the lowest AIC value was chosen. In cases where two models had similar AIC values, the investigator determined the model to be retained. The goodness-of-fit of the final model was verified using the Hosmer–Lemeshow test. Prediction probabilities were gauged using c-statistics, and confidence intervals (CIs) were computed using the Wald method.

To analyze recovery using drainage methods, a Kaplan–Meier survival analysis was performed using the log-rank test. To assess the recovery patterns associated with fever and laboratory findings using drainage methods, we determined significant recovery rates following the day (D-day) the procedure was performed using the Kruskal–Wallis test. Statistical analysis was performed using Prism 9.3 (GraphPad Software, San Diego, CA, USA), with *p* < 0.05 indicating statistical significance.

## 3. Results

### 3.1. Patients Characteristics and Drainage Methods

Table 1 presents participants’ clinical characteristics according to PCN (*n* = 80) and RUS (*n* = 98) drainage methods. Significant between-group differences were observed for age (*p* = 0.001), BMI (*p* < 0.001), and hospital stay (*p* < 0.001), with younger age, higher BMI, and longer hospital stays observed in the PCN group (13.0 days, interquartile range [IQR]: 8.5–16.0) than in the RUS group (8.0 days, IQR: 6.0–12.0, *p* < 0.001). While surgery rates were comparable (PCN 76.2% vs. RUS 70.4%; *p* = 0.482), in-hospital surgery was more frequent in PCN (77.0% vs. 52.2%) and re-admission surgery was more frequent in RUS (23.0% vs. 47.8%; overall *p* = 0.006), which helps explain the longer index hospital stay in the PCN group. However, no significant differences were observed in sex, incidences of hypertension, DM, and stroke, visit type (emergency vs. outpatient), onset of fever, stone location, stone size, coexistence of renal stones, or procedure drainage location. Table S1 presents the selected antibiotics and microbiological results following the drainage methods. Table S2 presents the demographic, clinical, urolithiasis-related, and microbiological characteristics according to sex.

### 3.2. Factors Associated with Early Recovery

Table 2 presents univariate and multivariate logistic regression analysis results for factors of early recovery. In the univariate analysis, the female sex (odds ratio [OR]: 0.39, 95% Confidence interval (CI): 0.19–0.80; *p* = 0.01) and DM (OR: 2.54, 95% CI: 1.27–5.14; *p* = 0.009) showed negative and positive associations with early recovery, respectively. Moreover, microbiological variables, urine culture, culture from any site, cultured *E. coli*, and ESBL positivity showed no associations with early recovery. We compared the predictive power of interactions with the female sex (‘+’ indicates female sex × [microbiological variable]), including ‘female + culture from any site,’ ‘female + urine culture,’ and ‘female + cultured *E. coli*’, in univariate models with that of ‘female sex’ in the multivariate model. In the univariate analysis, ‘female + cultured *E. coli*’ significantly predicted early recovery (OR: 0.35, 95% CI: 0.12–0.83; *p* = 0.026). However, when ‘female sex’ was added to the multivariate model, no enhanced predictive capability was observed. Mid (OR: 4.4, 95% CI: 1.41–15.53; *p* = 0.014) and upper (OR: 3.49, 95% CI: 1.34–10.93; *p* = 0.017) stone locations, compared with lower ureteral stones, were associated with early recovery. Additionally, the presence of stones in both renal units (OR: 2.61, 95% CI: 1.1–6.28; *p* = 0.029) and a large stone size (10–20 mm) correlated with early recovery (OR: 3.05, 95% CI: 1.09–9.55; *p* = 0.042). In multivariate logistic regression, the female sex remained a significant predictor of delayed recovery (OR: 0.44, 95% CI: 0.20–0.95, *p* = 0.036). However, DM (OR: 2.26, 95% CI: 1.08–4.79; *p* = 0.031), as well as mid- (OR: 5.4, 95% CI: 1.65–20.1; *p* = 0.007) and upper-ureter (OR: 3.32, 95% CI: 1.24–10.63; *p* = 0.026) stone locations, were prognostic factors for early recovery. Tables S3 and S4 delineate the demographic, clinical, and urolithiasis characteristics, including the choice of antibiotics and microbiological results for early recovery. Initial empirical antibiotic distribution did not differ between the early- and non-early-recovery groups (*p* = 0.644), and the frequency of ESBL-producing organisms was also not significantly different (27.3% vs. 17.2%, *p* = 0.213) (Table S3). In the drainage-method comparison, the PCN group had a higher rate of empiric carbapenem initiation (31.2% vs. 17.3%) and a higher prevalence of ESBL-producing organisms (26.2% vs. 14.3%) than the RUS group (Table S1; *p* = 0.036 and *p* = 0.045, respectively). Although the early-recovery group had a longer index hospital stay (Table S3), this was explained by a higher rate of in-hospital definitive surgery in that group (86.4% vs. 67.9%, χ^2^ = 4.77, *p* = 0.029). Figure S1 illustrates the receiver operating characteristic curve for this multivariate model, with an area under the curve value of 0.718 (Hosmer–Lemeshow goodness-of-fit test *p* = 0.437 confirmed the model’s adequacy).

### 3.3. Inflammatory and Renal Function Trends After Decompression

**Figure 2:**
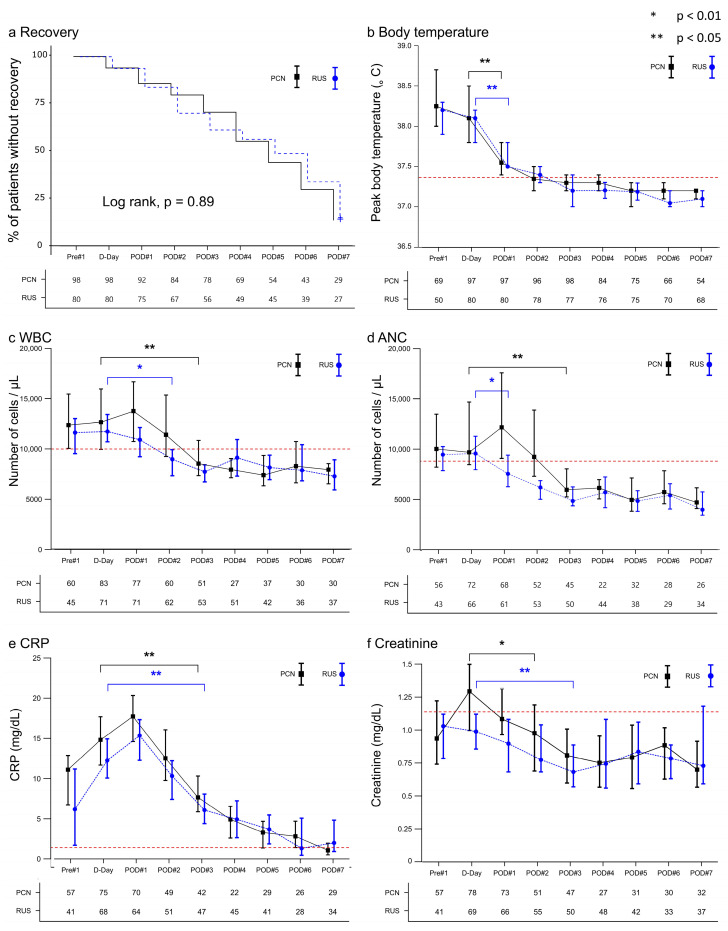
Comparison of the clinical course according to drainage procedures. (**a**) Kaplan–Meier curve representing the proportion of patients who did not achieve recovery according to each drainage method. (**b**) The peak value of fever on each respective day. (**c**) WBC count on each respective day. (**d**) ANC on each respective day. (**e**) CRP level on each respective day. (**f**) Creatinine level on each respective day. (**b**–**f**) represent changes in clinical parameters over time. In the corresponding whisker chart, the black square and blue circle depict the median values, and the ends of the whiskers indicate the 95% CI. D-day represents the day the drainage was performed, and each clinical parameter on the D-day was used as a reference to indicate the initial day of significant recovery among the subsequent days. WBC, White blood cell; ANC, Absolute neutrophil count; CRP, C-reactive protein; PCN, Percutaneous nephrostomy; RUS, Retrograde ureteral stent; CI, Confidence interval.

Figure 2 presents the clinical patterns of patients with SIRS following drainage via RUS and PCN. Early-recovery curves did not differ between PCN and RUS (log-rank *p* = 0.89, Figure 2a). Both drainage methods swiftly reduced the peak body temperatures on the first postoperative day (POD1, Figure 2b). The PCN group experienced elevated white blood cell [WBC] counts and absolute neutrophil counts [ANCs] immediately after the procedure, which recovered significantly on POD3 (Figure 2c,d). In contrast, their decline in the RUS group was greater. C-reactive protein [CRP] levels (Figure 2e) showed a comparable reduction on POD3 in both groups. Figure 2f shows a slightly significant recovery in the serum creatinine [Cr] value in the RUS group one day later than that in the PCN group and a significant recovery on POD3.

## 4. Discussion

Our cohort shows the post-decompression phase of recovery. In this context, factors traditionally linked to progression from OPN to SIRS (DM, larger stone size, more proximal stone location) showed positive associations with early recovery after the obstruction was relieved [14,15,16]. We interpret this as obstruction-dependent pathophysiology: while obstruction persists, elevated intrarenal pressure and infected urine amplify bacterial stimuli; decompression removes this driver, and systemic inflammation can resolve more rapidly in patients with more factors related to obstruction-dependent pathophysiology. By contrast, female sex was associated with slower recovery, consistent with greater systemic susceptibility rather than an obstruction-dependent pathophysiology. Initial antibiotic class and ESBL status were not associated with early recovery, supporting the hypothesis that early recovery was driven mainly by baseline clinical factors after decompression.

In the obstruction-dependent pathophysiology versus systemic susceptibility context, sex-linked evidence is as follows. Sex differences in stone disease and UTIs require attention to incidence and complication profiles. Although kidney stones are more common in males [1,2], stone-obstructive SIRS is reported more often in females [3,17], indicating greater UTI susceptibility in females. In broader sepsis cohorts, males show higher risks of sepsis and mortality [18,19], and often require more intensive care unit support [20,21]. while sepsis sources differ by sex: respiratory disease is common in males and UTIs are common in females [22,23]. In UTI-related sepsis and bacteremia, *E. coli* predominates [24], while it is isolated more frequently in females, reinforcing a sex-linked pathogen profile. In our cohort, females had higher positivity in any culture, urine culture, and *E. coli* isolation, with a trend toward higher positive blood cultures, suggesting a higher urinary pathogen burden at presentation despite SIRS-based enrollment. Adding microbiological variables to the female-sex variable did not improve prediction beyond sex alone. Given the high prevalence of *E. coli* in females, the association with SIRS in our cohort supports female sex as a risk factor for both infection and heightened systemic susceptibility rather than a marker of obstruction-dependent pathophysiology. Anatomical factors may contribute beyond pathogen identity [25]. By contrast, although DM is associated with systemic immune status [26], in emergently drained obstructive UTI, our data indicated that the DM interaction was linked to localized, obstruction-dependent processes that resolved after decompression.

From 1999 to 2009, a period marked by a 95.6% increase in calculus-induced OPN, differences in recovery patterns according to drainage methods indicated their potentially significant role in managing systemic inflammation [3]. Although the necessity for drainage has been increasing in these patients, the choice between RUS and PCN remains inconclusive [11,12,13]. In this study, no significant impact on early recovery was observed when comparing PCN with RUS. The baseline values were similar between both groups, with the PCN group showing increased WBC counts and ANC and delayed recovery compared with the RUS group. Urologists have proposed a theoretical risk of sepsis exacerbation due to elevated kidney pelvic pressure during RUS. No decisive evidence or guideline supports this claim; however, RUS does not result in a higher incidence of bacteremia, unlike PCN [27,28]. These findings may suggest that penetrating the pyelonephritic renal parenchyma leads to higher levels of inflammatory processes than those post-RUS. Importantly, the recovery durations for fever and CRP were comparable between the two groups. A marginally swifter recovery of kidney function was observed in the PCN group. In our cohort, patients managed with PCN were more likely to receive empiric carbapenems at presentation, a pattern consistent with hemodynamic instability (severe sepsis/septic shock) or recent isolation of ESBL-producing organisms. Despite this higher-risk profile and the more frequent use of carbapenems in the PCN cohort, neither the drainage method nor the initial antibiotic selection was significantly associated with early recovery. Therefore, the choice of the drainage method should be tailored to the patient’s medical status and specific clinical considerations.

Treatment guidelines should stratify patients to guide perioperative control of systemic inflammation in elective stone surgery. Mortality after URS for ureteric calculi is largely attributable to urosepsis and has increased in recent series [6,7]. Infection confined to the renal unit may progress to systemic infection, and higher intrarenal pressure during endoscopy may increase this risk. The clinical risk factors identified in this study likely also operate in post-URS sepsis. In our cohort, DM aligned with obstruction-related pathophysiology during drainage rather than with persistent SIRS severity; once SIRS is controlled, DM alone should not mandate prolonged preoperative treatment. A higher pathogen burden in females indicates a more severe infectious state and supports stricter perioperative protocols in females. These points are consistent with prior evidence showing that renal pelvis urine obtained before percutaneous nephrolithotomy predicts postoperative SIRS when bacterial growth is present [29]. On this basis, in stone-obstructive SIRS, collecting and culturing renal pelvis urine during drainage may better predict outcomes than bladder urine cultures. Prolonged obstruction exacerbates systemic inflammation and prolongs recovery [15,16]. The risk of sepsis after URS increases when a stent has been in place for more than one month; therefore, careful treatment timing is warranted [30].

The limitations of this study include its retrospective, single-center design and the issue that patient allocation was not comparable between the drainage methods. Obesity was more common in patients who underwent RUS than in those who underwent PCN, potentially biasing toward RUS due to the technical difficulties of PCN, influenced by skin-to-kidney distance. This could increase obesity-related complications in the RUS group. The preference for RUS in younger patients may introduce bias owing to better immune status. Excluding laboratory results of discharged patients may have biased the remaining patients’ results toward worse outcomes, although discharge timing and fever subsidence did not vary during the study.

## 5. Conclusions

Female sex independently predicted failure to achieve early recovery after urgent decompression, whereas DM and proximal stone location independently predicted early recovery. RUS and PCN achieved comparable early recovery rates; PCN was associated with a transient rise in leukocyte counts and earlier creatinine recovery. Consistent with an obstruction-dependent mechanism, early recovery was not related to initial antibiotic class or ESBL status and was associated with obstruction-related clinical factors (DM and proximal stone location) once the obstruction was relieved, with an inverse association for female sex. Management should be tailored to patient-specific clinical factors to optimize outcomes in stone-induced SIRS after decompression.

## Figures and Tables

**Table 1:** Demographic, clinical, and urolithiasis characteristics of patients according to drainage methods.

Variable	Total (n = 178)	PCN (n = 80)	RUS (n = 98)	*p*-Value
Sex, n (%)				0.470
Female	125 (70.2)	54 (67.5)	71 (72.4)	
Male	53 (29.8)	26 (32.5)	27 (27.6)	
Age, years, median [IQR]	68.5 [58.0–77.0]	73.0 [61.5–80.5]	66.0 [57.0–74.0]	0.001
BMI, kg/m^2^, median [IQR]	24.5 [21.7–27.7]	23.5 [20.3–25.9]	25.9 [22.9–29.0]	<0.001
HTN, n (%)	116 (65.2)	56 (70.0)	60 (61.2)	0.222
DM, n (%)	67 (37.6)	34 (42.5)	33 (33.7)	0.226
Stroke, n (%)	17 (9.6)	9 (11.2)	8 (8.2)	0.483
Visit type, n (%)				0.189
Emergency room	169 (94.9)	78 (97.5)	91 (92.9)	
Outpatient	9 (5.1)	2 (2.5)	7 (7.1)	
Hospital days, median [IQR]	10.5 [7.0–15.0]	13.0 [8.5–16.0]	8.0 [6.0–12.0]	<0.001
Prior to other hospital treatment, n (%)	55 (30.9)	24 (30.0)	31 (31.6)	0.823
Onset of fever, n (%)				0.123
1–3 days	146 (82.0)	72 (90.0)	74 (75.5)	
3 days–1 week	16 (9.0)	4 (5.0)	12 (12.2)	
~1 week	4 (2.3)	1 (1.2)	3 (3.0)	
Unknown	12 (6.7)	3 (3.8)	9 (9.2)	
Location of stone, n (%)				0.268
Low-ureter	47 (26.4)	24 (30.0)	23 (23.5)	
Mid-ureter	32 (18.0)	16 (20.0)	16 (16.3)	
Upper-ureter	92 (51.7)	39 (48.8)	53 (54.1)	
Kidney	7 (3.9)	1 (1.2)	6 (6.1)	
Size of stone, n (%)				0.616
approximately 5 mm	34 (19.1)	13 (16.2)	21 (21.4)	
5–9 mm	87 (48.9)	41 (51.2)	46 (46.9)	
10–20 mm	43 (24.2)	18 (22.5)	25 (25.5)	
>20 mm	14 (7.9)	8 (10.0)	6 (6.1)	
Coexistence of renal stones, n (%)				0.491
Free	75 (42.1)	31 (38.8)	44 (44.9)	
Both	40 (22.5)	19 (23.8)	21 (21.4)	
Ipsilateral	41 (23.0)	22 (27.5)	19 (19.4)	
Contralateral	22 (12.4)	8 (10.0)	14 (14.3)	
Surgery performed, n (%)	131 (73.6)	61 (76.2)	69 (70.4)	0.482
Timing of definite surgery, n (%)				0.006
Re-admission surgery	47 (36.2)	14 (23.0)	33 (47.8)	
In-hospital surgery	83 (63.8)	47 (77.0)	36 (52.2)	
Drainage location, n (%)				0.342
Right	80 (44.9)	36 (45.0)	44 (44.9)	
Left	91 (51.1)	39 (48.8)	52 (53.1)	
Both	7 (3.9)	5 (6.2)	2 (2.0)	

PCN, Percutaneous nephrostomy; RUS, Retrograde ureteral stenting; IQR, Interquartile range; BMI, Body mass index; HTN, Hypertension; DM, Diabetes mellitus.

**Table 2:** Univariate and multivariate logistic regression models of early recovery.

Variables	Univariate		Multivariate	
	OR (95% CI)	*p*-Value	OR (95% Wald CI)	*p*-Value
Age	1.02 (0.99–1.04)	0.217		
Female sex	0.39 (0.19–0.80)	0.01	0.44 (0.20–0.95)	0.036
+ culture from any positive sites	0.58 (0.29–1.15)	0.118		
+ urine positive culture	0.72 (0.36–1.42)	0.341		
+ *E. coli* positive culture	0.35 (0.12–0.83)	0.026		
HTN	1.37 (0.67–2.95)	0.397		
DM	2.54 (1.27–5.14)	0.009	2.26 (1.08–4.75)	0.031
Stroke	2.35 (0.8–6.55)	0.106		
BMI	0.95 (0.88–1.01)	0.13		
Emergency visit	0.37 (0.02–2.08)	0.35		
Microbiology				
Urine culture positive	1.67 (0.80–3.74)	0.189		
Culture from any positive sites	1.55 (1.20–2.79)	0.654		
*E. coli* positive culture	1.06 (0.54–2.10)	0.864		
ESBL positive	1.81 (0.79–3.99)	0.147		
RUS (vs. PCN)	0.6 (0.3–1.19)	0.142		
Stone location				
Low-ureter	Reference			
Mid-ureter	4.4 (1.41–15.53)	0.014	5.4 (1.58–18.52)	0.007
Upper-ureter	3.49 (1.34–10.93)	0.017	3.32 (1.15–9.55)	0.026
Kidney	1.4 (0.07–10.92)	0.775	1.48 (0.14–15.99)	0.747
Stone size				
0–5 mm	Reference			
5–10 mm	1.13 (0.42–3.41)	0.812		
10–20 mm	3.05 (1.09–9.55)	0.042		
~20 mm	1.87 (0.41–8)	0.401		
Coexistence of renal stone				
Free	Reference			
Both	2.61 (1.1–6.28)	0.029		
Ipsilateral	1.41 (0.55–3.51)	0.468		
Contralateral	1.28 (0.37–3.9)	0.674		

OR, Odds ratio; CI, Confidence interval; *E. coli*, *Escherichia coli*; DM, Diabetes mellitus; HTN, Hypertension; BMI, Body mass index; ESBL, Extended-spectrum beta-lactamases; RUS, Retrograde ureteral stenting; PCN, Percutaneous nephrostomy. ‘+’ indicates the interaction ‘female sex × [corresponding microbiological variable].

## Data Availability

The data related to this study are available within this article or Supplementary Materials.

## Supplementary Materials

The following supporting information can be downloaded at https://www.mdpi.com/article/10.3390/diagnostics15172282/s1. Figure S1: Receiver operating characteristic curve for predictors of early recovery according to multivariate logistic regression analysis; Table S1: Selection of antibiotics and microbiological results according to drainage methods; Table S2: Demographic, clinical, urolithiasis, and microbiological characteristics of patients according to sexes; Table S3: Demographic, clinical, and urolithiasis characteristics of patients according to early recovery; Table S4: Choice of antibiotics and microbiological results according to early recovery.
