# Supplementary material for: Diagnostic Clinical Predictors of Early Recovery from Stone-Induced Systemic Inflammatory Response Syndrome After Urgent Decompression
Source: Diagnostics (Basel). 2025 Sep 8;15(17):2282. doi: 10.3390/diagnostics15172282 (PMC12428519; doi:10.3390/diagnostics15172282)

Supplementary material

Table of Contents

Particulars	Page number
Table S1	2
Table S2	4
Table S3	6
Table S4	8
Figure S1	9

Table S1. Selection of antibiotics and microbiological results according to drainage methods.

Variables	Total (n=178)	PCN (n=80)	RUS (n=98)	p value
Initial antibiotics, n (%)				0.036
3rd generation cephalosporin	99 (55.6)	36 (45.0)	63 (64.3)	
Carbapenem	42 (23.6)	25 (31.2)	17 (17.3)	
Quinolones	32 (18.0)	15 (18.8)	17 (17.3)	
2nd generation cephalosporin	2 (1.1)	1 (1.2)	1 (1.0)	
Others	3 (1.7)	3 (3.8)	0 (0.0)	
Culture of any site, n (%)				0.549
Positive	133 (74.7)	62 (77.5)	71 (72.4)	
Negative	45 (25.3)	18 (22.5)	27 (27.6)	
Urine culture, n (%)				0.517
Positive	119 (66.9)	56 (70.0)	63 (64.3)	
Negative	59 (33.1)	24 (30.0)	35 (35.7)	
Blood culture, n (%)				0.177
Positive	78 (43.8)	40 (50.0)	38 (38.8)	
Negative	100 (56.2)	40 (50.0)	60 (61.2)	
Organism, n (%)				0.176
No growth	45 (25.8)	18 (22.5)	27 (27.6)	
E. coli	87 (48.3)	35 (43.8)	52 (53.1)	
K. pneumoniae	12 (6.7)	7 (8.8)	5 (5.1)	
Enterococcus spp.	6 (3.4)	2 (2.5)	4 (4.1)	
Proteus spp.	16 (9.0)	11 (13.8)	5 (5.1)	
Others	12 (6.7)	7 (8.7)	5 (5.1)	
ESBL, n (%)				0.045
Positive	35 (19.7)	21 (26.2)	14 (14.3)	
Negative	143 (80.3)	59 (73.8)	84 (85.7)	
Elective stone treatment, n (%)				0.125
Medical	48 (27.0)	19 (23.8)	29 (29.6)	
URS	37 (20.8)	19 (23.8)	18 (18.4)	
RIRS	42 (23.6)	16 (20.0)	26 (26.5)	
URS + RIRS	29 (16.3)	15 (18.8)	14 (14.3)	

PNL	7 (3.9)	6 (7.5)	1 (1.0)	
ESWL	15 (8.4)	5 (6.2)	10 (10.2)	
Blood transfusion, n (%)				0.594
Positive	6 (3.4)	4 (5.0)	2 (2.0)	
Negative	172 (96.6)	76 (95.0)	96 (98.0)	

Abbreviations: PCN, percutaneous nephrostomy; RUS, retrograde ureteral stenting; E. coli, Escherichia coli; K. pneumoniae, Klebsiella pneumoniae; ESBL, extended-spectrum beta-lactamase; URS, ureteroscopy; RIRS, retrograde intrarenal surgery; PNL, percutaneous nephrolithotomy; ESWL, extracorporeal shock wave lithotripsy.

Table S2. Demographic, clinical, urolithiasis, and microbiological characteristics of patients according to sexes.

Variable	Total (n=178)	Female (n=125)	Male (n=53)	p value
**Age,** years, median [IQR]	68.5 [58.0–77.0]	68.0 [57.0–78.0]	69.0 [58.0–76.0]	0.951
**BMI,** kg/m^2, median [IQR]	24.5 [21.7–27.7]	25.4 [22.2–28.3]	23.4 [20.7–26.1]	0.042
**HTN,** n (%)	116 (65.2)	78 (62.4)	38 (71.7)	0.308
**DM,** n (%)	67 (37.6)	38 (30.4)	29 (54.7)	0.004
**Stroke,** n (%)	17 (9.6)	6 (4.8)	11 (20.8)	0.002
**Visit type**, n (%)		0		0.893
Emergency room	169 (94.9)	118 (94.4)	51 (96.2)	
Outpatient	9 (5.1)	7 (5.6)	2 (3.8)	
**Onset of fever,** n (%)				0.085
1–3 days	146 (82.0)	108 (86.4)	38 (71.7)	
3 days–1 week	16 (9.0)	8 (6.4)	8 (15.1)	
>1 week	4 (2.3)	2 (1.6)	2 (3.8)	
Unknown	12 (6.7)	7 (5.6)	5 (9.4)	
**Hospital days,** median [IQR]	10.5 [7.0–15.0]	10.0 [7.0–14.0]	12.0 [8.0–15.0]	0.351
**Prior to other hospital treatment,** n (%)	55 (30.9)	43 (34.4)	12 (22.6)	0.169
**Location of stone,** n (%)				0.222
Low-ureter	47 (26.4)	34 (27.2)	13 (24.5)	
Mid-ureter	32 (18.0)	26 (20.8)	6 (11.3)	
Upper-ureter	92 (51.7)	59 (47.2)	33 (62.3)	
Kidney	7 (3.9)	6 (4.8)	1 (1.9)	
**Size of stone,** n (%)				0.194
Approximately 5 mm	34 (19.1)	28 (22.4)	6 (11.3)	
5–9 mm	87 (48.9)	62 (49.6)	25 (47.2)	
10–20 mm	43 (24.2)	27 (21.6)	16 (30.2)	
>20 mm	14 (7.9)	8 (6.4)	6 (11.3)	

**Coexistence of renal stone,** n (%)				0.068
Free	75 (42.1)	59 (47.2)	16 (30.2)	
Both	40 (22.5)	22 (17.6)	18 (34.0)	
Ipsilateral	41 (23.0)	28 (22.4)	13 (24.5)	
Contralateral	22 (12.4)	16 (12.8)	6 (11.3)	
**RUS (vs. PCN),** n (%)	98 (55.1)	71 (56.8)	27 (50.9)	0.58
**Early recovery,** n (%)	44 (24.7)	24 (19.2)	20 (37.7)	0.015
**Initial antibiotics, n (%)**				0.710
3rd generation cephalosporin	97 (54.5)	67 (53.6)	30 (56.6)	
Carbapenem	42 (23.6)	32 (25.6)	10 (18.9)	
Quinolones	32 (18)	22 (17.6)	10 (18.9)	
Others[a]	7 (3.9)	4 (3.2)	3 (5.7)	
**Culture from any sites, n (%)**				0.001
Positive	133 (74.7)	103 (82.4)	30 (56.6)	
**Urine culture, n (%)**				0.006
Positive	119 (66.9)	92 (73.6)	27 (50.9)	
**Blood culture, n (%)**				0.119
Positive	78 (43.8)	60 (48)	18 (34)	
**ESBL, n (%)**				0.704
Positive	35 (19.7)	26 (20.8)	9 (17)	
**Organism, n (%)**				< 0.001
E. coli	87 (48.9)	77 (61.6)	10 (18.9)	
Proteus spp.	12 (6.7)	6 (4.8)	6 (11.3)	

Abbreviations: Interquartile range; BMI, Body mass index; HTN, Hypertension; DM, Diabetes mellitus; RUS, Retrograde ureteral stenting; PCN, Percutaneous nephrostomy; ESBL, Extended-spectrum beta-lactamase; *E. coli*, *Escherichia coli*; *K. pneumoniae*, *Klebsiella pneumoniae*; [a]: 2nd-generation Cephalosporin, Cephalosporin + Quinolone.

Table S3. Demographic, clinical, and urolithiasis characteristics of patients according to early recovery.

Variable	Total (n=178)	Early recovery (n=44)	Non-early recovery (n=134)	p value
Sex, n (%)				0.009
Male	53 (29.8)	20 (45.5)	33 (24.6)	
Female	125 (70.2)	24 (54.5)	101 (75.4)	
Age, median [IQR]	68.5 [58.0–77.0]	70.0 [59.0–80.0]	68.0 [57.0–75.0]	0.503
BMI, median [IQR]	24.5 [21.7–27.7]	22.7 [20.2–27.9]	25.1 [22.4–27.7]	0.111
HTN, n (%)	116 (65.2)	31 (70.5)	85 (63.4)	0.396
DM, n (%)	67 (37.6)	24 (54.5)	43 (32.1)	0.008
Stroke, n (%)	17 (9.6)	7 (15.9)	10 (7.5)	0.135
Visit type, n (%)				0.455
Emergency room	169 (94.9)	43 (97.7)	126 (94.0)	
Outpatient	9 (5.1)	1 (2.3)	8 (6.0)	
Onset of fever, n (%)				0.852
1–3 days	146 (82.0)	36 (81.8)	110 (82.1)	
3 days–1 week	16 (9.0)	5 (11.4)	11 (8.2)	
>1 week	4 (2.3)	1 (2.3)	3 (2.2)	
Unknown	12 (6.7)	2 (4.5)	10 (7.5)	
Hospital days, median [IQR]	10.5 [7.0–15.0]	12.0 [8.0–18.5]	9.0 [6.0–14.0]	0.021
Prior other hospital treatment, n (%)	55 (30.9)	14 (31.8)	41 (30.6)	1.000
Location of stone, n (%)				0.044
Low ureter	47 (26.4)	5 (11.4)	42 (31.3)	
Mid ureter	32 (18.0)	11 (25.0)	21 (15.7)	
Upper ureter	92 (51.7)	27 (61.4)	65 (48.5)	
Kidney	7 (3.9)	1 (2.3)	6 (4.5)	
Size of stone, n (%)				0.062
~5 mm	34 (19.1)	6 (13.6)	28 (20.9)	
5–9 mm	87 (48.9)	17 (38.6)	70 (52.2)	
10–20 mm	43 (24.2)	17 (38.6)	26 (19.4)	

~20 mm	14 (7.9)	4 (9.1)	10 (7.5)	
Coexistence of renal stone, n (%)				0.169
Free	75 (42.1)	14 (31.8)	61 (45.5)	
Both	40 (22.5)	15 (34.1)	25 (18.7)	
Ipsilateral	41 (23.0)	10 (22.7)	31 (23.1)	
Contralateral	22	5 (11.4)	17 (12.7)	
Drainage location, n (%)				0.610
Both	7 (3.9)	1 (2.3)	6 (4.5)	
Right	91 (51.1)	25 (56.8)	66 (49.3)	
Left	80 (44.9)	18 (40.9)	62 (46.3)	
RUS (vs PCN), n (%)	98 (55.1)	20 (45.5)	78 (58.2)	0.194

Abbreviations: IQR, interquartile range; BMI, body mass index; HTN, hypertension; DM, diabetes mellitus; RUS, retrograde ureteral stenting; PCN, percutaneous nephrostomy.

Table S4. Choice of antibiotics and microbiological results according to early recovery.

Variables	Total<br>(N=178)	Early recovery<br>(N=44)	Non-early recovery<br>(N=134)	p value
Initial antibiotics, n (%)				0.644
3rd generation cephalosporin	97 (54.5)	22 (50)	75 (56)	
Carbapenem	42 (23.6)	10 (22.7)	32 (23.9)	
Quinolones	32 (18)	9 (20.5)	23 (17.2)	
Othersa	7 (3.9)	3 (6.8)	4 (3)	
Culture of any sites, n (%)				0.803
Positive	133 (74.7)	34 (77.3)	99 (73.9)	
Urine culture, n (%)				0.255
Positive	119 (66.9)	33 (75.0)	86 (64.2)	
Blood culture, n (%)				0.669
Positive	78 (43.8)	21 (47.7)	57 (42.5)	
ESBL, n (%)				0.213
Positive	35 (19.7)	12 (27.3)	23 (17.2)	
Organism, n (%)				0.996
E. coli	87 (48.3)	22 (50)	65 (48.5)	
Proteus spp.	12 (6.7)	3 (6.8)	9 (6.7)	
K. pneumoniae	6 (3.4)	2 (4.5)	4 (3)	
Enterococcus spp.	16 (9)	4 (9.1)	12 (9)	
Others	12 (6.7)	3 (6.8)	9 (6.7)	
No growth	45 (25.8)	10 (22.7)	35 (26.1)	
Blood transfusion, n (%)				1.000
Positive	6 (3.4)	1 (2.3)	5 (3.7)	

Abbreviations: ESBL, extended-spectrum beta-lactamase; E. coli, Escherichia coli; K. pneumoniae, Klebsiella pneumoniae; Others—2nd-generation Cephalosporin, Cephalosporin + Quinolone.

Figure S1. Receiver operating characteristic curve for predictors of early recovery according to multivariate logistic regression analysis. ROC: receiving operating characteristic.

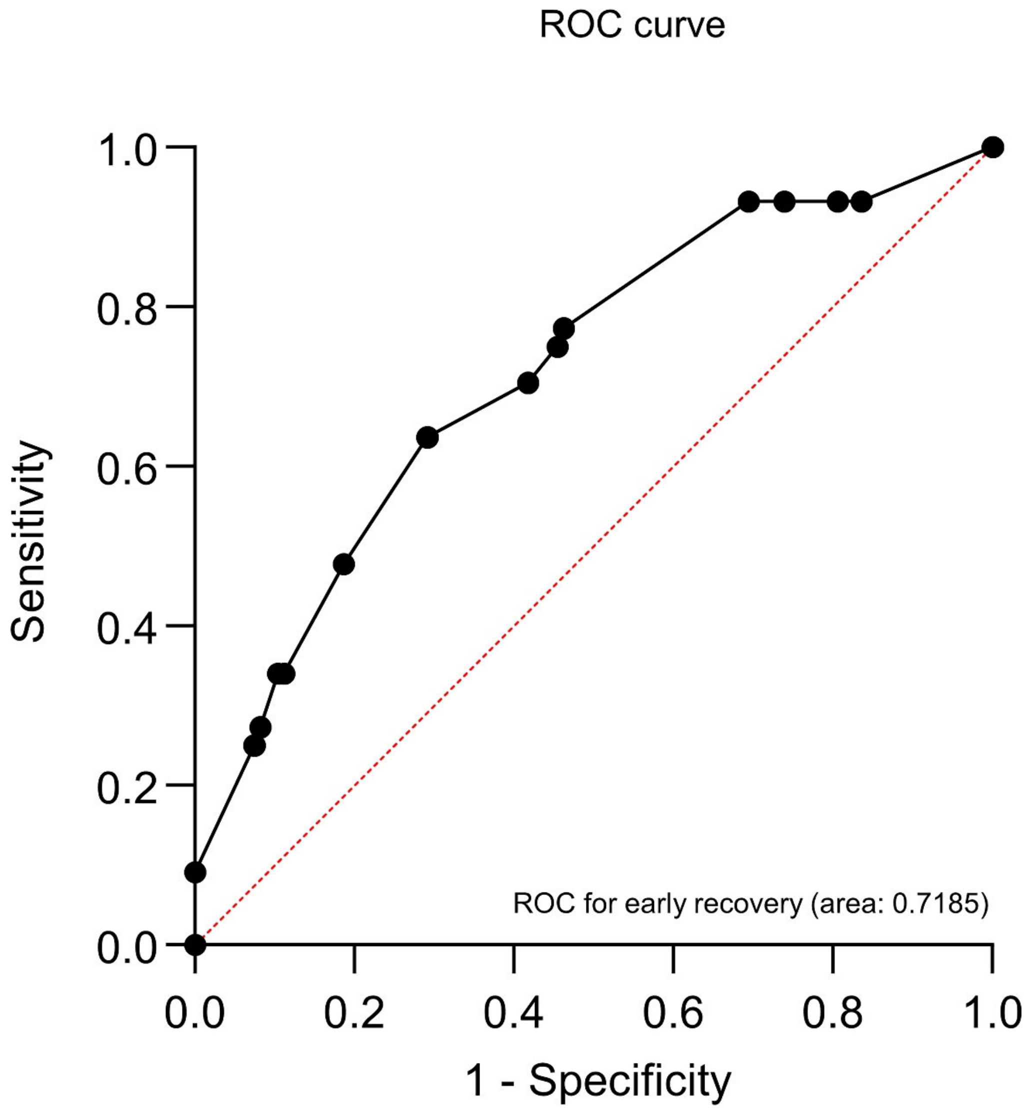

Supplement: Supplementary file 1 [file diagnostics-15-02282-s001.zip › diagnostics-3813682-supplementary.pdf]
